# Loss of a globally unique kelp forest from Oman

**DOI:** 10.1038/s41598-022-08264-3

**Published:** 2022-03-23

**Authors:** M. A. Coleman, M. Reddy, M. J. Nimbs, A. Marshell, S. A. Al-Ghassani, J. J. Bolton, B. P. Jupp, O. De Clerck, F. Leliaert, C. Champion, G. A. Pearson, E. A. Serrão, P. Madeira, T. Wernberg

**Affiliations:** 1 Present Address: National Marine Science Centre, New South Wales Fisheries, 2 Bay Drive, Coffs Harbour, NSW 2450 Australia; 2 National Marine Science Centre, Southern Cross University, 2 Bay Drive, Coffs Harbour, NSW 2450 Australia; 3 UWA Oceans Institute and School of Biological Sciences, University of Western Australia, 35 Stirling Highway, Crawley, WA 6009 Australia; 4 Department of Biological Sciences, University of Cape Town, Private Bag X3, Cape Town, 7701 South Africa; 5 Department of Marine Science and Fisheries, College of Agricultural and Marine Sciences, Sultan Qaboos University, Muscat, Oman; 6 Institute for Marine and Antarctic Studies, University of Tasmania, Hobart, Australia; 7 Fisheries Research Centre - Dhofar, Directorate General of Fisheries Research, Ministry of Agriculture, Fisheries and Water Resource, Salalah, Sultanate of Oman; 8 Senior Consultant – Marine, P.O. Box 389, Puerto Princesa, Palawan, 5300 Philippines; 9 Biology Department, Ghent University, Krijgslaan 281, Building S8, 9000 Ghent, Belgium; 10 Meise Botanic Garden, Nieuwelaan 38, 1860 Meise, Belgium; 11 CCMAR, CIMAR, University of Algarve, Gambelas, 8005-139 Faro, Portugal

**Keywords:** Biogeography, Conservation biology

## Abstract

Kelp forests are declining in many regions globally with climatic perturbations causing shifts to alternate communities and significant ecological and economic loss. Range edge populations are often at most risk and are often only sustained through localised areas of upwelling or on deeper reefs. Here we document the loss of kelp forests (*Ecklonia radiata*) from the Sultanate of Oman, the only confirmed northern hemisphere population of this species. Contemporary surveys failed to find any kelp in its only known historical northern hemisphere location, Sadah on the Dhofar coast. Genetic analyses of historical herbarium specimens from Oman confirmed the species to be *E. radiata* and revealed the lost population contained a common CO1 haplotype found across South Africa, Australia and New Zealand suggesting it once established through rapid colonisation throughout its range. However, the Omani population also contained a haplotype that is found nowhere else in the extant southern hemisphere distribution of *E. radiata*. The loss of the Oman population could be due to significant increases in the Arabian Sea temperature over the past 40 years punctuated by suppression of coastal upwelling. Climate-mediated warming is threatening the persistence of temperate species and precipitating loss of unique genetic diversity at lower latitudes.

## Introduction

Climate change has precipitated loss and decline in kelp forests in many regions globally, threatening the vast ecological and economic values that these systems underpin^1,2^. Warming and extreme events have driven contemporary loss of kelp forests and transitions to alternate ecosystem states e.g.^3–7^. Marginal kelp populations at warm, equatorward range edges are often among the most vulnerable to climate change because they experience temperatures near or exceeding their thermal thresholds and may lack the genetic diversity to respond^8^. Such kelp populations inhabiting warm seascapes are often only able to persist due to localised regions of cooler water produced by upwelling or thermoclines at depth that create refugia from warming e.g.^9–11^. Concerningly, these marginal populations often harbour unique^12,13^ or functional^14,15^ genetic diversity.

The dominant and most widespread kelp in the southern hemisphere is *Ecklonia radiata* (C. Agardh) J. Agardh, which is also among the most warm-tolerant kelps^16^. This species has a shallow phylogeographic history with low genetic diversity^17,18^ suggestive of a recent evolutionary origin. Indeed, the genus *Ecklonia* may have crossed the equator from the northern Pacific < 5 mya^19^ and rapidly colonised southern temperate reefs^17,20^ during a period of cooling. The only northern hemisphere population of *E. radiata* that has been studied is in the Sultanate of Oman, hereafter Oman^21^. This population has been documented and studied in situ in a series of unpublished reports and herbarium specimens from the 1980’s^22,23^ from a single location on the southern Dhofar coastline (Sadah; 17.0420°N, 55.0796°E). Although possible northern hemisphere populations of *E. radiata* have been reported from Mauritania, Senegal and the Canary Islands^16,21^ and there are unconfirmed reports of *E. radiata* from Socotra, Yemen^24^, these reports are unreliable or unconfirmed. For example, inspection of photographs of the putative *E. radiata* in Socotra^24^ revealed a morphologically entirely distinct species that is unlikely to be *E. radiata*. Moreover, the Atlantic Ocean Mauritania and Canary Islands specimens have never been seen or reported in situ and are recorded as *E. muratii* on herbarium specimens. Reports of *E. radiata* in Senegal (Cabo Verde, a tropical archipelago) are most likely mislabelled samples from Cape Vert (Senegal, an upwelling zone; E.A. Serrão pers. obs.). Hence, we consider Oman to be the only likely northern hemisphere population of *E. radiata* but its identity remains to be confirmed genetically.

As with many low latitude kelp forests, the Omani population of *E. radiata* is thought to be sustained by localized cool water upwelling that allows this species to persist in an otherwise warm seascape. Intense upwelling occurs 20 km either side of Sadah^23^, where high nutrient levels and low temperatures (as low as 15.9 °C) facilitated the persistence of *E. radiata* populations between the months of July to mid-October^25,26^. As temperatures rise to > 27 °C in the post-monsoonal season (and occasionally > 30 °C), *E. radiata* sporophytes at Sadah senesce, adopting an annual life history^22,23^. Senescence and adoption of an annual life history has also been speculated to occur in *E. radiata* at the Abrolhos Islands off Western Australia^27^, the warmest location this species occurs in Australia. The adoption of an annual life history may occur because temperature tolerances of sporophytes, but not gametophytes, are exceeded in warmer months^16,28^.

Over the past few decades, the Dhofar coast has warmed and there have been periods of suppressed upwelling^29–32^. Although periodic suppression of upwelling and deepening of thermoclines can lead to temporary loss of kelp canopy cover^33^, the low latitude position of the Oman kelp forest, where summer temperatures often exceed the upper thermal limits of *E. radiata* sporophytes^16^ and the lack of nearby populations from which reefs could be reseeded following loss, renders this isolated population of *E. radiata* vulnerable to extinction. Thus, we conducted the first targeted surveys off the remote Sadah coast since the 1980s to confirm the contemporary presence of *E. radiata*. Our failure to find any extant *E. radiata* off Oman led us to analyse historical *E. radiata* herbarium specimens from Sadah to (a) confirm the species identity, (b) elucidate pathways of colonization and (c) compare patterns of genetic diversity with extant populations from throughout the Indian Ocean (Southern Africa and Western Australia).

## Methods

### Historical data and surveys

**Figure 1:**
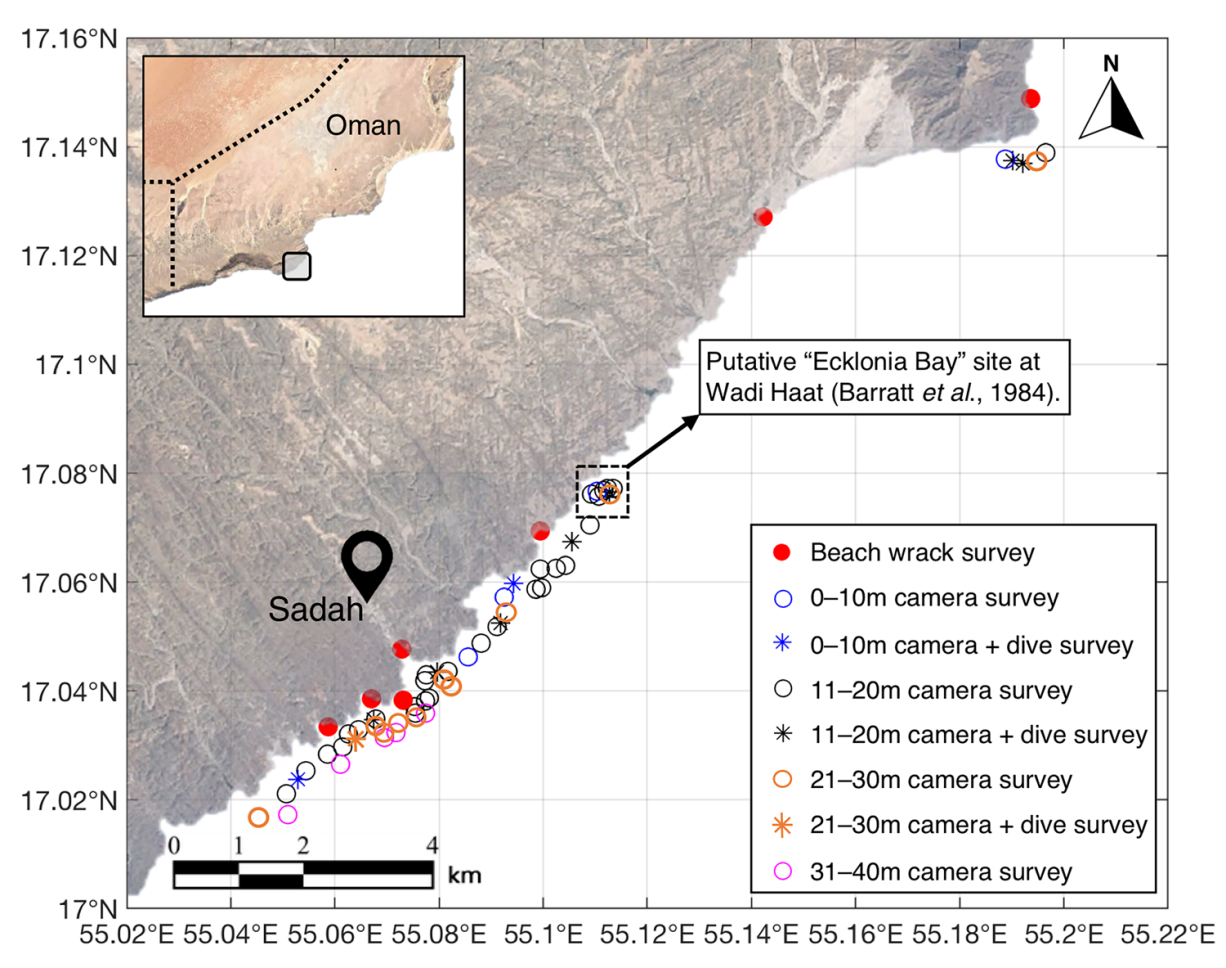
Spatial distribution of sites surveyed (*n* = 64) throughout the Sadah region of south-western Oman to assess the contemporary occurrence of *Ecklonia radiata*. Map generated using MATLAB (ver. 9.8, The MathWorks, Inc., Natick, MA, USA, https://au.mathworks.com/products/matlab).

The only documented evidence of *E. radiata* from Oman are by^34^, who first noted the presence of *E. radiata* in 1982 during surveys of the distribution of the starfish *Acanthaster planci* in Dhofar, and by^22,23^. In particular, detailed studies (including temporal studies on productivity and herbivory) were conducted in 1983 and 1985 at a site they called ‘*Ecklonia* Bay’ at Wadi Haat, (misspelt as Haart), Sadah (17.0420°N, 55.0796°E, Fig. 1), which was the only place the authors found *E. radiata* from a number of sites along the southern Dhofar coast. This record of *E. radiata* was published in a short abstract on the Dhofar macroalgal community^35^.

The Sadah *Ecklonia* site from^22,23^ was characterised as being bedrock with gullies extending to a depth of 20 m and approximately 200 m offshore, with large outcrops of bedrock on sand down to 30 m. Details of *E. radiata* surveys at these sites can be found in^22,23^, but briefly, *Ecklonia* forests were present at this one location at depths of 6.5–20 m, apparently below a thermocline. These surveys reported *E. radiata* as the dominant species at this site, especially between 9 and 20 m. These plants were mostly stage 2 sporophytes^36^ with an average length of 60–70 cm in the immediate post-monsoon period (mid-September). *Ecklonia* plants were found down to 30 m in densities similar to shallower waters but only a few plants reached stage 3 and these were mostly found below 20 m. Data on biomass, productivity and other parameters for *Ecklonia* at 12 m depth were measured in 1985 and found to be comparable with published data from similar depths for *E. radiata* in Australasia^16^. Seasonal growth data were collected and presumed to be maximal in July–August and slow to almost zero by November. The entire *Ecklonia* sporophyte population was reported to disappear in the monsoonal period by late January/early February as temperatures rose to and often exceeded 27 °C^22^.

### Contemporary surveys

Targeted subtidal surveys for *E. radiata* (by Coleman & Wernberg) were done off the Sadah coast in October 2019 in and around the only site where *E. radiata* was found in^23^ (Fig. 1). The exact location of the single *E. radiata* population studied^23^ was only known from rough hand drawn maps and local site names, so locating the exact site was subjective. Hence, the putative Waadi Haat “*Ecklonia* Bay” site was surveyed as well as a range of sites and depths around the Sadah coast based on the maps, descriptions and names provided in the reports as well as additional sites to the north and south. Over a 5-day period in the middle of the post-monsoonal season when *E. radiata* was previously documented to be at peak abundance^22,23^ we conducted towed video surveys, SCUBA dives and shore-based surveys of wrack ~ 15 km either side of Sadah and between 1 and ~ 30 m depth in an attempt to locate *E. radiata* (Fig. 1). The middle of the post-monsoonal period was targeted because even if there had been shifts in phenology of this annual *E. radiata* population since the 1980s, this would most likely still have captured its presence in surveys. 57 towed video surveys of ~ 5 min in duration each were conducted (~ 200–400 m each). Towed video footage was watched in real time and also recorded to be viewed later and any potential *E. radiata* noted. We also conducted 10 dives across a range of sites and depths (chosen from video footage) to search for *E. radiata*. On each dive, we thoroughly searched for *E. radiata* along a 6 m wide belt on 30 m transects (n = 2), roving swimming surveys (~ 20 min each dive) and photographed the benthos in *n* = 30, 25 × 25 cm photo quadrats for later analysis and inspection for *E. radiata*. Finally, we conducted 6 × 1-h roving beach wrack surveys at different sites along the Sadah coast to search for *E. radiata* wrack (washed up sporophytes) which would show presence of *E. radiata* from previous years, seasons or unsampled sites.

### Genetic analyses of historical and contemporary samples

**Figure 2:**
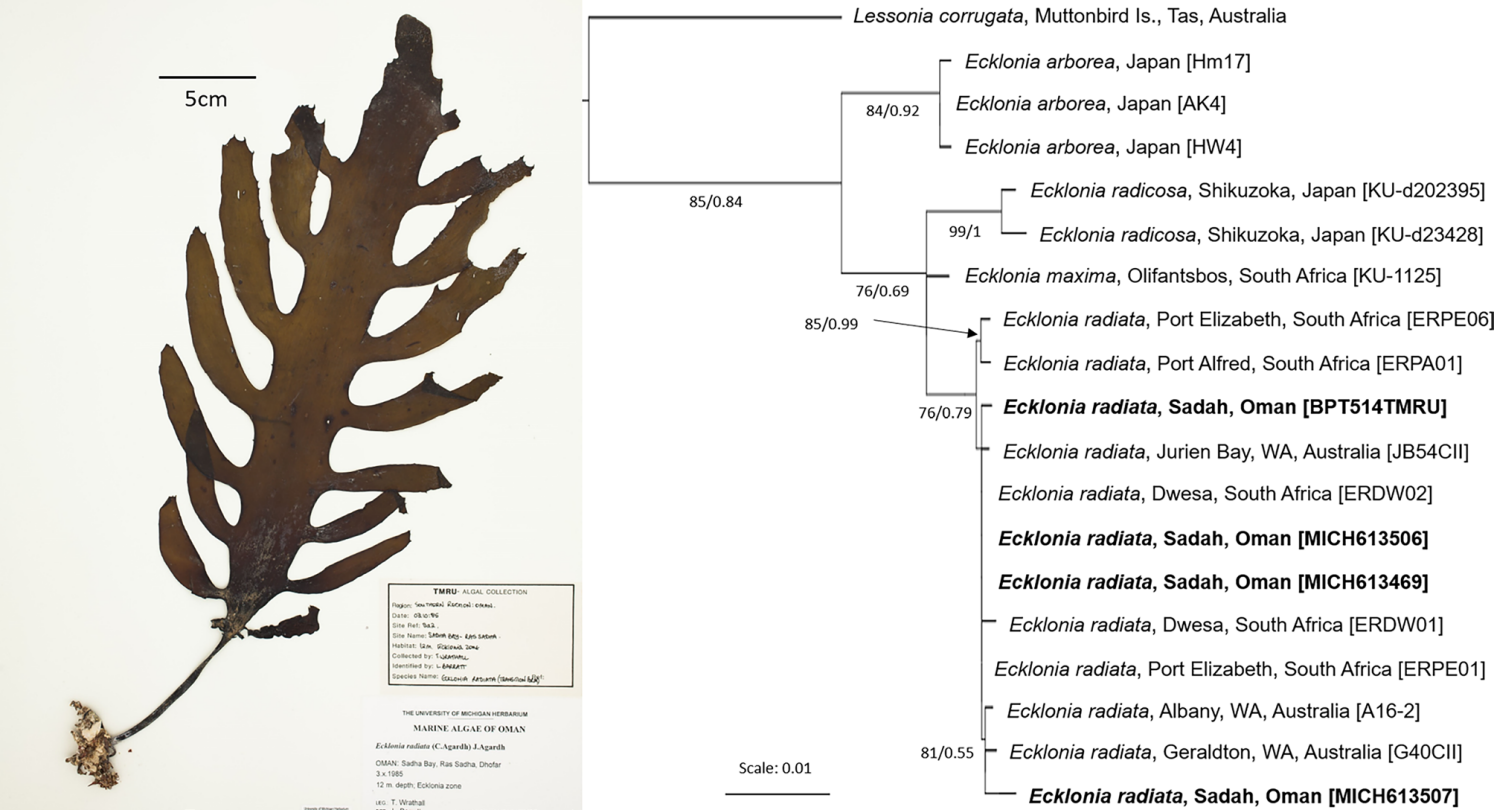
Example herbarium specimen of *E. radiata* from Oman collected in the 1980s (MICH613469, high resolution photograph available at https://macroalgae.org/portal/) and molecular phylogeny of *Ecklonia* rooted with *Lessonia corrugata* showing clear clustering of Omani *Ecklonia* sequences (in bold) within *E. radiata* confirming its species identity*.* Topology and branch lengths from maximum likelihood (ML) analysis of concatenated COI, Trnw1, Atp8 and rbcL sequences. Values provided as approximate Bayesian support (right) and ML bootstrap (left), with some intraclade values removed for clarity. DNA isolate numbers are provided to allow comparison with the text. Scale represents average number of nucleotide substitutions per site. See Supplementary Table 1 for Genbank accession numbers.

Specimens of *E. radiata* from Sadah (collected in 1985 and 1987 by T. Wrathall and L. Barratt) deposited in herbaria of the University of Michigan (MICH613469, MICH613507 (n = 2 recruits), MICH613506; Fig. 2, high resolution photographs available at https://macroalgae.org/portal/), Sultan Qaboos University (SQUH00006214, TMRU #514) and the Oman Marine Science and Fisheries Centre, Ministry of Agriculture and Fisheries (MSFC#138) were accessed and shipped to the National Marine Science Centre in Australia. For these herbarium specimens, DNA was extracted from approximately 50 mg tissue from each sample using the combined CTAB and SDS protocol^37^. Extracted DNA was purified using a Mo-Bio PowerClean Pro DNA clean-up kit (Merck KGaA, Darmstadt, Germany) following the manufacturer’s protocol. Purified DNA was PCR amplified in 20 µL volumes using 0.5 µL of purified DNA extract in an Eppendorf Mastercycler Nexus Gradient thermocycler using the Thermo Scientific Phire Plant Direct PCR Master Mix kit (Thermo Fisher Scientific, Inc. Waltham, Massachusetts, USA), following the manufacturer’s ‘dilute and store’ protocol. Only 4 of these herbarium specimens were able to be amplified and sequenced.

We also used existing DNA extracted from fresh *E. radiata* material from Western Australia^14^ and dried material stored in silica gel from southern Africa to examine relationships among *E. radiata* from throughout the Indian Ocean. Specimens from Mozambique were collected from a reef off the coast of Zavora, Inhambane Province at 34 m depth at Tentação (24.4444°S, 35.3589°E). Voucher specimens are deposited in the herbarium of Meise Botanic Garden (www.botanicalcollections.be). For the southern African material, dried seaweed samples were ground into a fine powder using a Tissue lyser II (Qiagen, Hilden, Germany). Genomic DNA was extracted using the NucleoSpin® 96 Tissue kit (Macherey–Nagel, Duren, Germany) and diluted (1:100) before PCR amplification. For samples from Western Australia, DNA was extracted and cleaned as in^14^. All methods were performed in accordance with relevant guidelines and regulations and samples were collected under scientific collection permits issued by state or federal governments and herbarium specimens obtained under agreements with each herbarium/institution. Collection permits were RES2016/02 (Southern Africa), 6210/10/53 for Oman and CE005834, SW019697, #2050 and #3349 (Western Australia).

To confirm the species identity of Omani herbarium specimens, we amplified four DNA markers (COI, trnW-1, atp8 and *rbcL*) for a small subset of Australian, east African and Omani material to compare to published phylogenetic studies of *Ecklonia* (see supplementary material Table 1 for Genbank accession numbers). Two mitochondrial intergenic spacer regions (trnW-1 and atp8) were amplified using the primers trnW-trn1-F (5′ GGGGTTCAAATCCCTCTCTT 3′), trnW-trn1-R (5′ CCTACATTGTTAGCTTCATGAGAA 3′) for the trnWI marker and atp8-trnS-F (5′ TGTACGTTTCATATTACCTTCTTTAGC 3′) and atp8-trnS-R (5′ TAGCAAACCAAGGCTTTCAAC 3′) for the atp8 marker^38^. The RuBisCO large subunit (*rbcL*) chloroplast marker was amplified using the primers KL2 (5′ GATGCTGATTATAACGTTAAAG 3′) and KL8 (5′ GTTGGTGCATTTGACCACA 3′)^39^. Additionally, a portion of the mitochondrial *cox*1-5´ barcoding region (COI) was amplified using the previously published primers Gaz F2 (5′ CCAACCAYAAAGATATWGGTAC 3′) and Gaz R2 (5′ GGATGACCAAARAACCAAAA 3′)^39^ for all material (southern African, Australian and Omani). In accordance with the Thermo Scientific Phire Plant Direct PCR Master Mix kit manufacturer instructions, a gradient series PCR was performed, using purified DNA, to determine the optimal annealing temperature for each primer/template pair (southern African material: 55.6 °C and Australia/Oman material: 53.5 °C for Gaz F2 and Gaz R2; 53.0 °C for atp8-trnS-F and atp8-trnS-R; 53.0 °C for trnW-trn1-F and trnW-trn1-R; and 48.0 °C for KL2 and KL8). For each marker, PCR cycling consisted of an initial denaturation for 5 min at 98 °C, followed by 45 cycles of: 5 s at 98 °C, 5 s at the relevant annealing temperature for the primer/template pair and 45 s at 72 °C. A final extension was carried out for 1 min at 72 °C. Visualisation of PCR products was carried out using a 2% agarose gel and successful amplicons were purified and sequenced at the Australian Genomic Research Facility (AGRF), Sydney or the Central Services and Technologies of CCMAR, Portugal. Sequences were de novo assembled using Geneious Prime 11.1.5 (Biomatters Ltd., Auckland, New Zealand)^40^, and edited by eye. Primers were trimmed from alignments, and data quality checks were carried out with MegaBLAST (NCBI, Bethesda, Maryland, USA)^41^, and for the COI marker, protein translation. Concatenated and single gene alignments and a partition file (gene length) were imported into W-IQ-Tree for tree reconstruction^42^. W-IQ-Tree incorporates ModelFinder^43^, which automatically selects and applies a best-fit model to each partition^44^, which, in this study was identified as the HKY + F + I, F81 + F, TN + F and F81 + F models for the COI, trnW-1, atp8 and *rbcL* partitions respectively. W-IQ-Tree provides ultrafast bootstrap (BS)^45^ and  approximate Bayesian branch support values. The resultant tree was visualised using FigTree 1.4.4 (Edinburgh, Scotland, UK)^46^ and rooted with *Lessonia corrugata*^18^.

To examine patterns of genetic diversity and relationships among *E. radiata* samples from throughout the Indian Ocean we amplified the CO1 marker from the full Indian Ocean dataset (*n* = 79; Table 1) as described above. CO1 haplotype number (H), diversity (*Hd*) and nucleotide diversity (π) were calculated using DnaSP version 6.12.03 (Barcelona, Catalonia, Spain)^47^. Haplotype TCS networks^48^ were reconstructed using PopArt (Dunedin, New Zealand)^49^. New sequences were deposited in Genbank under accession numbers in Supplementary Table 1.

**Table 1:** CO1 diversity metrics for the *E. radiata* population in Oman compared to extant Western Australian and Southern African populations.

	Pops	N	K	S	Pi	H	Hd
Oman	1	4	0.500	1	0.00081	2	0.500
Western Australia	4	43	0.412	2	0.00069	3	0.401
Southern Africa	6	32	1.748	4	0.00284	4	0.708
All regions		79	1.334	7	0.00225	7	0.586

Pops, number of populations sampled; N, total sample size; k, average number of nucleotide differences; S, number of segregating sites; Pi, nucleotide diversity; H, number of haplotypes; Hd, haplotype diversity (corrected for sample size).

### Assessment of historical environmental conditions

To investigate potential changing environmental conditions on *E. radiata* off Sadah, variables known to influence the physiology and distribution of kelp were quantified throughout a 20 km radius surrounding *E. radiata’s* historical occurrence off Sadah (17.0420°N, 55.0796°E) from January 1982 to January 2019. These included daily sea surface temperature (SST) from the reprocessed (level 4) Operational SST and Ice Analysis system (OSTIA) with 0.05° spatial resolution and the direction (i.e., positive, neutral or negative) and intensity of the Indian Ocean Dipole (IOD) mode index for the period January 1982 to January 2019. The IOD mode index is a measure of the gradient in SST between the western and eastern equatorial Indian Ocean. This climate-driver affects environmental conditions throughout the Indian Ocean, with a positive IOD mode associated with anomalously warm ocean temperatures and the suppression of upwelling in the western Indian Ocean including the coastal environment off Oman^31^. SST data were downloaded from the Copernicus Marine Environment Monitoring Service (https://marine.copernicus.eu; SST product #010_011) and IOD mode index data were downloaded from the National Oceanic and Atmospheric Administration’s Physical Sciences Laboratory (https://psl.noaa.gov/gcos_wgsp/Timeseries/DMI).

Time-series of SST and IOD mode index were plotted to assess for potential environmental drivers of change in *E. radiata* occurrence off Sadah between historical and contemporary surveys. Linear models were fitted to minimum, mean and maximum annual SST data to test for ocean warming trends off Sadah between January 1982 and January 2019. Plotting and analysis of environmental data was undertaken using the software R R Core^50^, with residual plots assessed visually to confirm that linear models fitted to SST data satisfied the assumptions of normality and homogeneity of variance.

### Additional information

All samples were collected under scientific collection permits issued by state or federal governments in each country and herbarium specimens obtained under agreements with each herbarium/institution. Scientific collection permits were RES2016/02 (Southern Africa), 6210/10/53 for Oman and CE005834, SW019697, #2050 and #3349 (Western Australia).

## Results

### Failure to find extant E. radiata populations in Oman

We did not find any evidence of extant populations of *E. radiata* in Oman following 5 days of surveys off Sadah in the peak post-monsoon season, the period it was reported to be abundant in all surveys from the 1980s. This included 57 towed video surveys and 10 SCUBA dives at depths it was reported to be historically abundant around Sadah (Fig. 1). Moreover, surveys at deeper depths and on reefs surrounding Sadah also did not reveal any *E. radiata*. Moreover, no *E. radiata* was found during additional beach wrack surveys where washed up *E. radiata* plants may have accumulated from previous seasons or years or deeper depths (Fig. 1). Notably, since these 2019 surveys, Omani scientists have confirmed the absence of *E. radiata* at Sadah. They assessed benthic habitats (~ 200m^2^ per site) in 3 areas off Dhofar including Haat at Sadah, as part of a juvenile abalone seeding project. These surveys were done over 7 days in October/November 2020 for the abundance of macroalgae generally, but no *Ecklonia* was seen (S.A. Al-Ghassani pers. Obs.).

### Species identification & genetic diversity

After trimming primer sequences, PCR amplification and sequencing yielded 655 bp of COI, 326 bp of trnW-1, 167 bp of atp8 and 1044 bp of *rbcL*. MegaBLAST searches of the NCBI database identified best matches for all sequences from the Omani specimens to be sequence data of *E. radiata.* This hypothesis was further supported by a phylogenetic reconstruction based on maximum likelihood (ML) using the concatenated dataset (COI, trnW-1, atp8 and *rbcL*), which recovered these sequences within a well-supported (BS = 99) monophyletic clade comprising *E. radiata* sequence data from samples spanning its range (Fig. 2).

**Figure 3:**
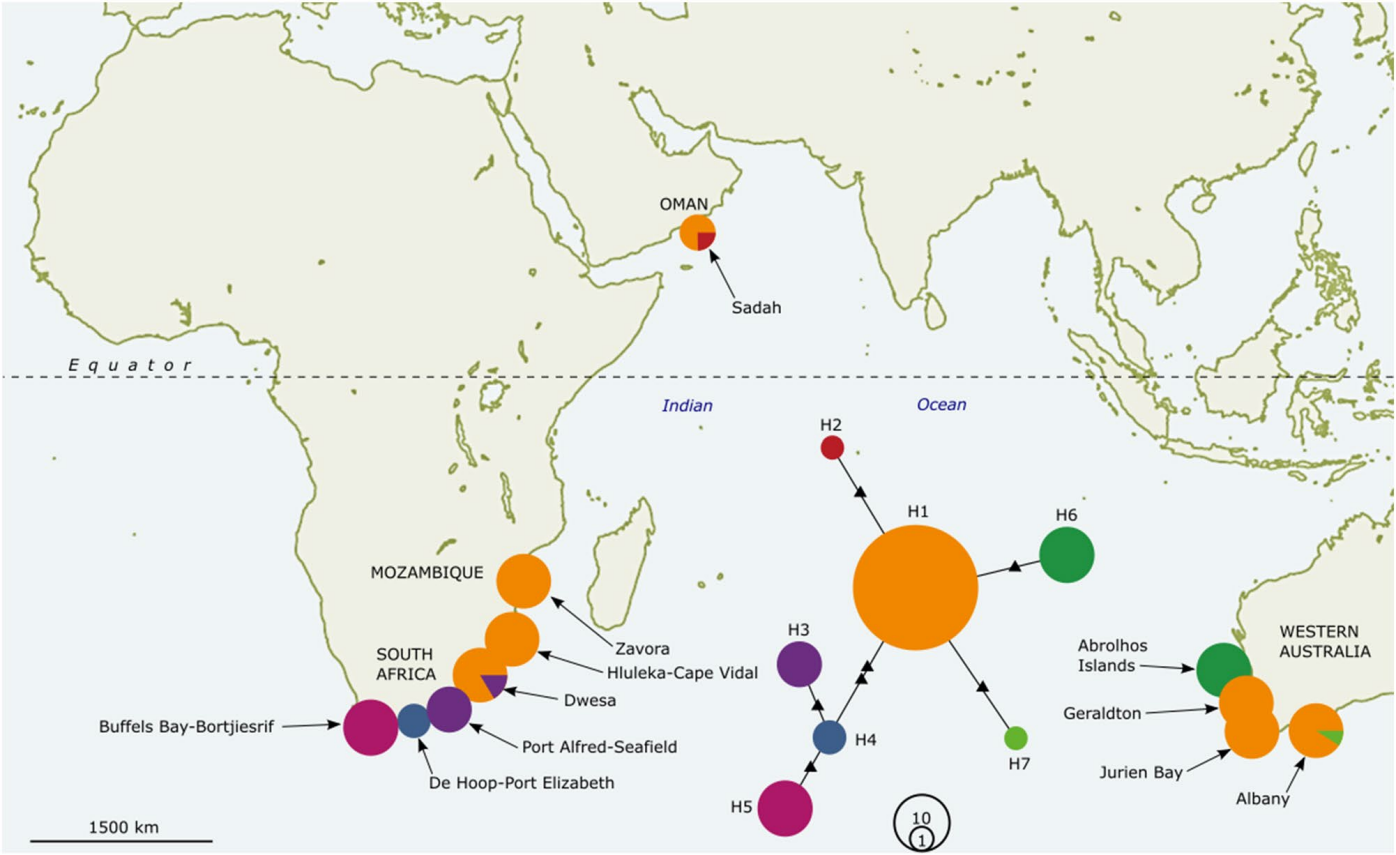
CO1 haplotype network of *E. radiata* from Oman and throughout the Indian Ocean. Colours represent different haplotypes and circle size represents sample size. See Supplementary Table 1 for Genbank accession numbers. Map created in Inkscape v1.1.1 (https://inkscape.org/release/inkscape-1.1.1/).

CO1 haplotype diversity (*H* = 0.586) was much greater than nucleotide diversity (π = 0.00225) indicative of only minor nucleotide differences between haplotypes (Table 1). A total of seven COI haplotypes were present among *E. radiata* samples from the Indian Ocean (H1-7, Table 1). A single widely distributed haplotype (H1) was found across all three major regions of the Indian Ocean (Oman, Australia and Africa) (Fig. 3). All regions had unique haplotypes including Oman, which had 1 unique haplotype (H2) from the 4 specimens we were able to amplify (Fig. 3). Haplotype diversity (Hd = 0.5) was high in Oman relative to other regions, despite only 4 individuals being able to be genotyped (Table 1).

### Trends and anomalies in environmental conditions

**Figure 4:**
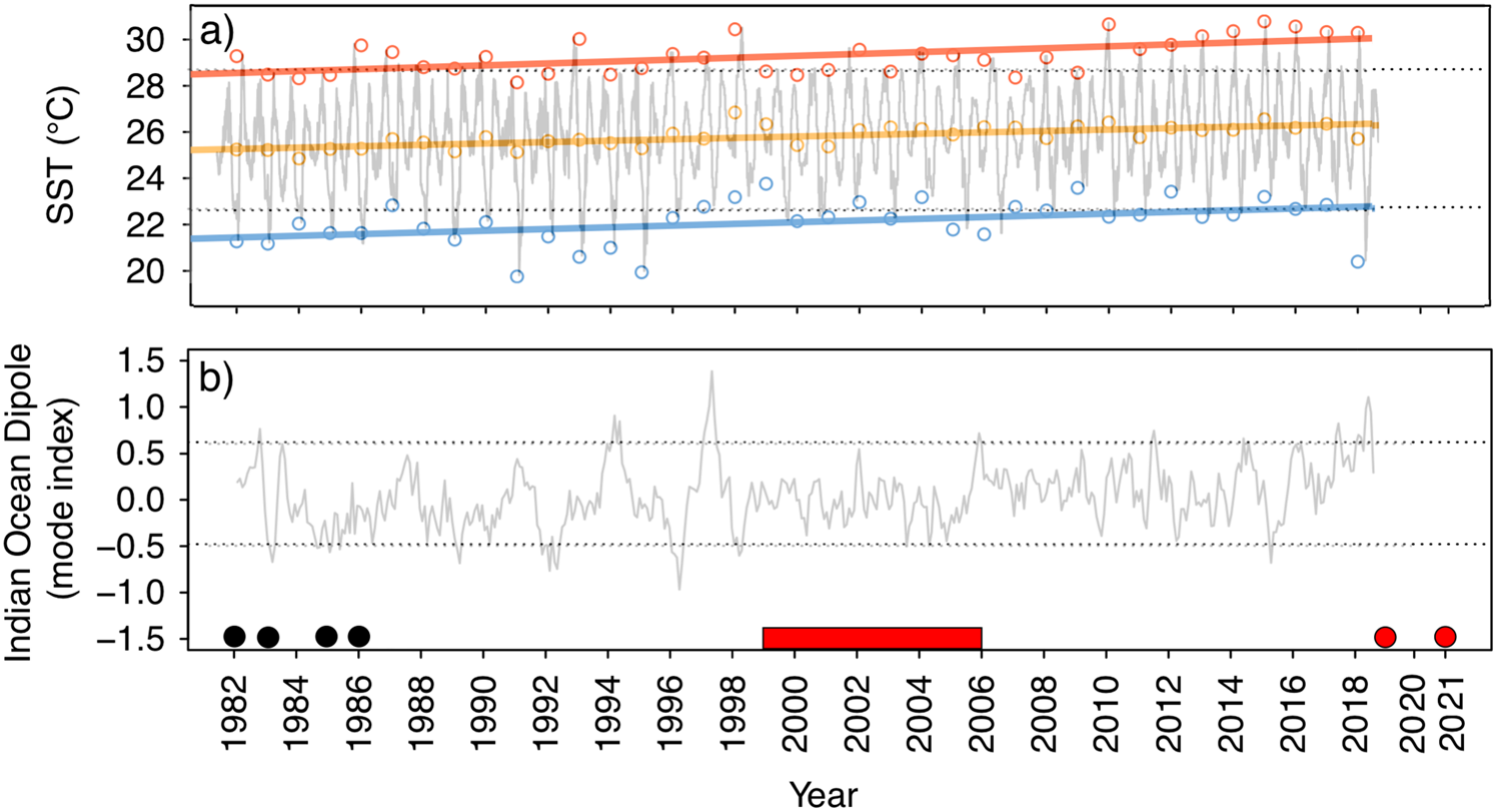
Time-series of satellite-derived (**a**) sea surface temperature (SST) within a 15 km radius surrounding historical occurrences of *Ecklonia radiata* off Sadah, Oman (17.0420°N, 55.0796°E) and (**b**) the intensity of the Indian Ocean Dipole (IOD) mode index, which is determined by the gradient in SST between the western equatorial Indian Ocean (50°E–70°E and 10°S–10°N) and the eastern equatorial Indian Ocean (90°E–110°E and 10°S–10°N). N.B. strong positive IOD mode indices are associated with anomalously warm ocean temperatures and the suppression of upwelling in the Western Indian Ocean, including coastal environments off Oman. Trends in minimum (blue data), mean (orange data) and maximum (red data) annual SST have been overlaid in panel a), which show significant (at alpha level 0.05) positive increases in minimum (+ 1.36 °C ± 1.01 °C 95% CI), mean (+ 1.12 °C ± 0.39 °C 95% CI) and maximum (+ 1.52 °C ± 0.72 °C 95% CI) SST from 1982 to 2018 throughout the spatial extent assessed. Dashed horizontal lines on all plots denote 90% confidence intervals for each time-series. Black circles on panel B represent dates when *E. radiata* was documented in Oman and red lines and circles represent dates of surveys when it was not seen in surveys.

Minimum, mean and maximum annual SST was found to have significantly increased off Sadah, Oman, between 1982 and 2018 (min: *F*_1,35_ = 7.14, *P* = 0.01, + 1.36 °C ± 1.01 °C 95% CI; mean: *F*_1,35_ = 34.07, *P* < 0.001, + 1.12 °C ± 0.39 °C 95% CI; max: *F*_1,35_ = 17.76, *P* < 0.001, + 1.52 °C ± 0.72 °C 95% CI; Fig. 4a). The IOD index consistently varied between positive and negative mode between January 1982 and January 2019 (Fig. 4b). However, notable positive IOD events occurred during 1994 and 1997, which were associated with an intensity that exceeded all other positive or negative IOD events throughout the time-period analysed.

## Discussion

Marginal, warm edge populations of kelp are declining in many regions globally^3,6,51^ threatening the unique genetic diversity such marginal populations often support^13,14,52^. Here, we report the loss of the *E. radiata* population from Oman, the only confirmed northern hemisphere population of this species. Of concern is that this local extinction implies the loss of haplotype diversity that is found nowhere else in the species’ extant range. Significant ocean warming trends punctuated by periods of suppressed upwelling that supported the existence of this species in a shallow, low latitude seascape, are likely to have driven the loss of this kelp population.

### Warming, upwelling and kelp loss

It is apparent that *Ecklonia radiata* was thriving off Sadah, Oman in the 1980s^22,23^ but was not found in contemporary surveys. Although there had not been any targeted surveys done at Sadah since the 1980s, the only site that reported *E. radiata* off the Dhofar coast^23^, several other non-targeted surveys (e.g. for fish, abalone or taxonomy) were done at other sites but did not report any *E. radiata*. For example, the Oman Seaweed Project (OSP; 1998/99), surveyed seaweeds off Dhofar but did not find any *E. radiata*. Similarly, the Algal Biodiversity Project of Oman (1999–2002) led to several publications^53,54^, but also did not report *E. radiata*. Tom Schils and Klaas Pauly of the Phycology Research Group, Ghent University, Belgium, carried out surveys mainly around Masirah Island and Barr Al Hikman on the Arabian Sea and the Sea of Oman coastlines over the period 1999 to 2006 e.g.^55–57^. Although the latter publication lists *Ecklonia radiata* in Dhofar this is presumed to be the record from^54^ which is listed in their references. None of these authors found any evidence of *E. radiata* in Oman. Most notable, however, is that the local Omani scientists and abalone divers that regularly work along the Dhofar coastline conducting surveys for abalone where *E. radiata* could be present, have not seen any *E. radiata* over the past few decades (pers. comm. S A Al.-Ghassani). Hence, although many of the above surveys did not specifically visit Sadah or look for *E. radiata,* it appears that this species was likely lost from Oman many decades ago.

While we cannot definitively determine the cause of *E. radiata* loss from Sadah, we suggest that long-term ocean warming in the region, punctuated by short-term environmental events that have substantially altered the physical environment, such as periodic cessation of upwelling^31^, caused the documented extinction of *Ecklonia* off Oman. The Western Indian Ocean has been continually warming since the start of twentieth century, with the rate of warming accelerating during the last five decades^30^. Our primary data analysis specific to the coastal ocean off Sadah supports the gradual warming of this region, with maximum annual temperatures increasing by ~ 1.5 °C since 1982, to an average maximum of over 28 °C in 2019, which would have persistently challenged the upper thermal limit (26 °C) of *E. radiata*^16^.

Despite being a warm-tolerant kelp with an upper extreme thermal tolerance range between 21.2–26.5 °C in other parts of its range^16^, *E. radiata* is unlikely to have survived such temperatures. Warming would have caused the thermocline described by Barratt et al.^23^ at 6.5 m likely extended to deeper water + 15m^58^, to include the whole *E. radiata* forest including any fully grown (stage 3) plants. Therefore, ocean temperatures are likely to have exceeded the upper thermal limit of *E. radiata* sporophytes throughout the complete depth distribution of the population off Sadah. These high temperatures (i.e., >  ~ 28 °C and occasionally > 30 °C) are in excess of any known tolerances for both sporophytes and gametophytes in all other parts of the *E. radiata* distribution. Even if Omani kelp forests had a higher thermal tolerance than elsewhere in its range, such extreme temperatures in excess of 30 °C would likely have caused the loss of *E. radiata* from Oman. A similar disappearance was observed of an *Ecklonia cava* population in 1997 to 1999 in Tosa Bay, Japan, with only urchin barrens and coralline algae found in 2000^7,59^. Anomalously high sea surface temperatures recorded in this region during 1997–98 El Niño Southern Oscillation event were implicated in this disappearance of *E. cava*^7^. It is possible that *E. radiata* persists in Oman in much deeper (cooler) areas than what was sampled in the 1980s or the present study (> 40 m) and Autonomous Underwater Vehicle (AUV) surveys could clarify if this is the case. However, the complete lack of *Ecklonia* plants in our beach wrack surveys and the poor water clarity off Oman during monsoonal upwelling (and hence low light penetration at depth) makes this unlikely relative to other low latitude areas where *Ecklonia* persists in much deeper, clearer water e.g. 50–80m^9,60–62^.

### Genetic confirmation and possible origin of Omani *E. radiata*

Our analysis of historical herbarium specimens confirmed Omani kelp as *E. radiata* and represent the first genetic sequences for this population*.* We also reveal that its evolutionary origins are from a single common CO1 haplotype that is present throughout the southern hemisphere suggesting rapid historical colonization. However, the Omani population also contained a CO1 haplotype that is found nowhere else in the Indian (this paper) or Pacific Ocean (M.A. Coleman unpbl data) distribution of this species. Indeed, diversification from the common haplotype was found in all marginal populations sampled here (H3/4/5 from South Africa and H6 from the Abrolhos Islands, Western Australia) indicative of isolation following rapid colonisation e.g. Abrolhos^14^, and consistent with known genetic breaks^63^. The genetic diversity of *E. radiata* in Oman in the 1980s was likely higher than that reported here given that one unique haplotype was found from only 4 herbarium specimens (Hd = 0.5) which is higher than the diversity reported for extant Western Australian populations (Hd = 0.401, *n* = 43 specimens).

It is interesting to speculate on the origin of the Omani *E. radiata* population and how it may have crossed the equator. The presence of *E. radiata* populations on isolated islands (e.g. Lord Howe Island, Houtman Abrolous Islands) elsewhere within its range suggests that long distance dispersal is possible despite the lack of floating structures. Thus, we suggest that colonization in Oman may have been via “stepping stone” dispersal among cooler regions of upwelling (Somalia, Yemen, Oman) and/or cooler deep reefs in more tropical locations e.g. Mozambique^64,65^. Indeed, there are unconfirmed reports of deepwater *E. radiata* off Madagascar, and Sodwana Bay, South Africa (50 m, K. Sink Pers. Comm.), and in this study we confirmed the presence of the species in Inhambane, Mozambique (29–35 m) suggesting that this may have been a dispersal pathway during periods of cooling. Confirming this hypothesis could benefit from analysis of seaweed biogeography from other parts of the Arabian and African coast including Somalia where there is also significant monsoon-induced upwelling^56,66^.

An alternative hypothesis for the presence of a single, isolated population of *E. radiata* in Oman is an introduction from east Africa, potentially via historical frankincense^67^ or general maritime trade routes. The Omani empire was centred on the island of Zanzibar and maritime trade with East African ports further south such as Sofala on the coast of Mozambique and on Madagascar was common. The Omani vessels could have carried algal fragments from this region back to Oman after pulling up anchors or fishing gear. Indeed, stone ballast, anchors and pots associated with shipwrecks found in Zanzibar, Mombasa and Kilwa (Tanzania) are similar to those also found in Oman and other Arabian ports and date back to the ninth century^68,69^. The presence of a unique CO1 haplotype in Oman suggests that if this were the case, it was likely an old and single introduction followed by isolation or that the source population was not sampled (e.g. the unique haplotype may be present in unconfirmed/unsampled populations off Somalia or Yemen).

The *E. radiata* population in Oman was likely living on the edge, only sustained by the cooler waters that came with the localized monsoonal upwelling along this small section of the Dhofar coast. Globally, many isolated kelp populations persist at low latitudes only on deeper reefs where waters are cooler^9,10,12,70^ and further retreat to depth of kelp populations is predicted under future ocean warming^62^. Continued warming of our oceans and climate-induced change to oceanographic processes such as upwelling threatens these populations and the genetic diversity they can harbor. While it is unlikely that in situ conservation actions will be enough to protect these marginal populations against ongoing warming, we can take proactive measures to secure their diversity in germplasm or culture banks^71^. Protecting this diversity ex situ may one day present new opportunity to proactively restore or boost resilience of declining kelp forests^72^.

## Supplementary Information

Supplementary Information.

## Data Availability

Genbank accession numbers for CO1 sequences generated available in Supplementary Table 1.
