# Supplementary material for: Loss of a globally unique kelp forest from Oman
Source: Sci Rep. 2022 Mar 23;12:5020. doi: 10.1038/s41598-022-08264-3 (PMC8943203; doi:10.1038/s41598-022-08264-3)
Supplement: Supplementary file 1 — Supplementary Information. [file 41598_2022_8264_MOESM1_ESM.docx]

**Supplementary Table 1.** Details of sequenced specimens, museum supplied tissue samples and GenBank retrieved sequences data.

| **Species** | **Locality** | **Voucher/isolate** |  | | **GenBank Accession** | | | **Reference** |
| --- | --- | --- | --- | --- | --- | --- | --- | --- |
|  |  |  | **COI** | **Trnw1** | | ***Atp8*** | ***rbcL*** |  |
| *Lessonia corrugata* | Muttonbird Island, Tas, Australia | MuttonBirdIsland01 | KT158926 | - | | - | KT158887 | Durrant et al. 2015 |
| *Ecklonia arborea* | Japan | HW4 | LC146972 | - | | - | - | Yoshino et al. 2018 |
| *Ecklonia arborea* | Japan | AK4 | LC146968 | - | | - | - | Yoshino et al. 2018 |
| *Ecklonia arborea* | Japan | Hm17 | LC146952 | - | | - | - | Yoshino et al. 2018 |
| *Ecklonia radicosa* | Japan | KU-d23428 | LC493639 | - | | LC493676 | LC493745 | Kawai et al. 2020 |
| *Ecklonia cava* | Shizuoka, Japan | KU-d22395 | LC493635 | - | | LC493671 | LC493741 | Kawai et al. 2020 |
| *Ecklonia maxima* | Cape Town, South Africa | KU-1125 | LC493638 | - | | LC493674 | LC493744 | Kawai et al. 2020 |
| *Ecklonia radiata* | Oman | MICH613507 | OL456994 | OL457012 | | - | OL456995 | This paper |
| *Ecklonia radiata* | Oman | MICH613506 | OL456993 | OL457011 | | - | - | This paper |
| *Ecklonia radiata* | Oman | MICH613469 | OL456992 | OL457013 | | - | - | This paper |
| *Ecklonia radiata* | Oman | BPT514TMRU | OL456959 | OL457014 | | - | OL456997 | This paper |
| *Ecklonia radiata* | Abrolhos Islands, WA, Australia | AB1 | OL456948 | - | | - | - | This paper |
| *Ecklonia radiata* | Abrolhos Islands, WA, Australia | AB2 | OL456949 | - | | - | - | This paper |
| *Ecklonia radiata* | Abrolhos Islands, WA, Australia | AB3 | OL456950 | - | | - | - | This paper |
| *Ecklonia radiata* | Abrolhos Islands, WA, Australia | AB4 | OL456951 | - | | - | - | This paper |
| *Ecklonia radiata* | Abrolhos Islands, WA, Australia | AB5 | OL456952 | - | | - | - | This paper |
| *Ecklonia radiata* | Abrolhos Islands, WA, Australia | AB6 | OL456953 | - | | - | - | This paper |
| *Ecklonia radiata* | Abrolhos Islands, WA, Australia | AB7 | OL456954 | - | | - | - | This paper |
| *Ecklonia radiata* | Abrolhos Islands, WA, Australia | AB8 | OL456955 | - | | - | - | This paper |
| *Ecklonia radiata* | Abrolhos Islands, WA, Australia | AB9 | OL456956 | - | | - | - | This paper |
| *Ecklonia radiata* | Abrolhos Islands, WA, Australia | AB10 | OL456957 | - | | - | - | This paper |
| *Ecklonia radiata* | Jurien Bay, WA, Australia | JB54CII | OL456962 | OL457018 | | OL457005 | OL456999 | This paper |
| *Ecklonia radiata* | Jurien Bay, WA, Australia | JB10 | OL456982 | - | | - | - | This paper |
| *Ecklonia radiata* | Jurien Bay, WA, Australia | JB11 | OL456983 | - | | - | - | This paper |
| *Ecklonia radiata* | Jurien Bay, WA, Australia | JB15 | OL456984 | - | | - | - | This paper |
| *Ecklonia radiata* | Jurien Bay, WA, Australia | JB17 | OL456985 | - | | - | - | This paper |
| *Ecklonia radiata* | Jurien Bay, WA, Australia | JB18 | OL456986 | - | | - | - | This paper |
| *Ecklonia radiata* | Jurien Bay, WA, Australia | JB19 | OL456987 | - | | - | - | This paper |
| *Ecklonia radiata* | Jurien Bay, WA, Australia | JB21 | OL456988 | - | | - | - | This paper |
| *Ecklonia radiata* | Jurien Bay, WA, Australia | JB23 | OL456989 | - | | - | - | This paper |
| *Ecklonia radiata* | Jurien Bay, WA, Australia | JB26 | OL456990 | - | | - | - | This paper |
| *Ecklonia radiata* | Jurien Bay, WA, Australia | JB28 | OL456991 | - | | - | - | This paper |
| *Ecklonia radiata* | Geraldton, WA, Australia | G40CII | OL456961 | OL457019 | | OL457006 | OL456996 | This paper |
| *Ecklonia radiata* | Geraldton, WA, Australia | G1 | OL456972 | - | | - | - | This paper |
| *Ecklonia radiata* | Geraldton, WA, Australia | G37 | OL456973 | - | | - | - | This paper |
| **Species** | **Locality** | **Voucher/isolate** | **GenBank Accession** | | | | | **Reference** |
|  |  |  | **COI** | **Trnw1** | | ***Atp8*** | ***rbcL*** |  |
| *Ecklonia radiata* | Geraldton, WA, Australia | G39 | OL456974 | - | | - | - | This paper |
| *Ecklonia radiata* | Geraldton, WA, Australia | G45 | OL456975 | - | | - | - | This paper |
| *Ecklonia radiata* | Geraldton, WA, Australia | G49 | OL456976 | - | | - | - | This paper |
| *Ecklonia radiata* | Geraldton, WA, Australia | G51 | OL456977 | - | | - | - | This paper |
| *Ecklonia radiata* | Geraldton, WA, Australia | G52 | OL456978 | - | | - | - | This paper |
| *Ecklonia radiata* | Geraldton, WA, Australia | G53 | OL456979 | - | | - | - | This paper |
| *Ecklonia radiata* | Geraldton, WA, Australia | G56 | OL456980 | - | | - | - | This paper |
| *Ecklonia radiata* | Geraldton, WA, Australia | G60 | OL456981 | - | | - | - | This paper |
| *Ecklonia radiata* | Albany, WA, Australia | A16 | OL456958 | - | | - | - | This paper |
| *Ecklonia radiata* | Albany, WA, Australia | A17 | OL456963 | - | | - | - | This paper |
| *Ecklonia radiata* | Albany, WA, Australia | A18 | OL456964 | - | | - | - | This paper |
| *Ecklonia radiata* | Albany, WA, Australia | A19 | OL456965 | - | | - | - | This paper |
| *Ecklonia radiata* | Albany, WA, Australia | A20 | OL456966 | - | | - | - | This paper |
| *Ecklonia radiata* | Albany, WA, Australia | A21 | OL456967 | - | | - | - | This paper |
| *Ecklonia radiata* | Albany, WA, Australia | A22 | OL456968 | - | | - | - | This paper |
| *Ecklonia radiata* | Albany, WA, Australia | A23 | OL456969 | - | | - | - | This paper |
| *Ecklonia radiata* | Albany, WA, Australia | A24 | OL456970 | - | | - | - | This paper |
| *Ecklonia radiata* | Albany, WA, Australia | A26 | OL456971 | - | | - | - | This paper |
| *Ecklonia radiata* | Albany, WA, Australia | A16-2 | OL456960 | OL457021 | | OL457008 | OL457002 | This paper |
| *Ecklonia radiata* | Zavora, Mozambique | MOZT03 | OM650143 |  | |  |  | This paper |
| *Ecklonia radiata* | Zavora, Mozambique | MOZDRS03 | OM650144 |  | |  |  | This paper |
| *Ecklonia radiata* | Zavora, Mozambique | MOZDR03 | OM650145 |  | |  |  | This paper |
| *Ecklonia radiata* | Kei Mouth, South Africa | D1759 | - | KM575831 | | KM575732 | - | Rothman et al. (2017) |
| *Ecklonia radiata* | Cape of Good Hope, South Africa | EXBB13 | OM650135 |  | |  |  | This paper |
| *Ecklonia radiata* | Mkhambati, South Africa | ERMK03 | OM650123 |  | |  |  | This paper |
| *Ecklonia radiata* | Mkhambati, South Africa | ERMK02 | OM650124 |  | |  |  | This paper |
| *Ecklonia radiata* | Mkhambati, South Africa | ERMK01 | OM650125 |  | |  |  | This paper |
| *Ecklonia radiata* | Dwesa, South Africa | ErDIV01 | OM650132 |  | |  |  | This paper |
| *Ecklonia radiata* | Dwesa, South Africa | ERDM09 | OM650131 |  | |  |  | This paper |
| *Ecklonia radiata* | Dwesa, South Africa | ERDW01 | OM650130 |  | |  |  | This paper |
| *Ecklonia radiata* | Dwesa, South Africa | ERDW02 | OM650129 |  | |  |  | This paper |
| *Ecklonia radiata* | Dwesa, South Africa | ERDW04 | OM650128 |  | |  |  | This paper |
| *Ecklonia radiata* | Hluleka, South Africa | D1769 | - | KM575813 | | KM575813 | - | Rothman et al. (2017) |
| *Ecklonia radiata* | Hluleka, South Africa | D1771 | - | KM575816 | | KM575816 | - | Rothman et al. (2017) |
| *Ecklonia radiata* | Hluleka, South Africa | D1770 | - | KM575818 | | KM575818 | - | Rothman et al. (2017) |
| *Ecklonia radiata* | Hluleka, South Africa | ERHLU02 | OM650126 |  | |  |  | This paper |
| *Ecklonia radiata* | Hluleka, South Africa | ERHLU01 | OM650127 |  | |  |  | This paper |
| *Ecklonia radiata* | Cape Vidal, South Africa | ERVID01 | OM650134 |  | |  |  | This paper |
| *Ecklonia radiata* | Buffels Bay, South Africa | EXBB05 | OM650135 |  | |  |  | This paper |
| *Ecklonia radiata* | Buffels Bay, South Africa | EXBB02 | OM650136 |  | |  |  | This paper |
| **Species** | **Locality** | **Voucher/isolate** | **GenBank Accession** | | | | | **Reference** |
|  |  |  | **COI** | **Trnw1** | | ***Atp8*** | ***rbcL*** |  |
| *Ecklonia radiata* | Bortjiesrif, South Africa | ERBOT01 | OM650139 |  | |  |  | This paper |
| *Ecklonia radiata* | Bortjiesrif, South Africa | ERBOT02 | OM650138 |  | |  |  | This paper |
| *Ecklonia radiata* | Bortjiesrif, South Africa | ERBOT03 | OM650137 |  | |  |  | This paper |
| *Ecklonia radiata* | Bortjiesrif, South Africa | EHBOT01 | OM650142 |  | |  |  | This paper |
| *Ecklonia radiata* | Bortjiesrif, South Africa | EHBOT02 | OM650141 |  | |  |  | This paper |
| *Ecklonia radiata* | Bortjiesrif, South Africa | EHBOT03 | OM650140 |  | |  |  | This paper |
| *Ecklonia radiata* | De Hoop, South Africa | D1737 | - | KM575738 | | KM575801 | - | Rothman et al. (2017) |
| *Ecklonia radiata* | De Hoop, South Africa | ERSDH03 | OM650133 |  | |  |  | This paper |
